# Use of Technology and Its Association With Academic Performance and Life Satisfaction Among Children and Adolescents

**DOI:** 10.3389/fpsyt.2021.764054

**Published:** 2021-11-11

**Authors:** Saray Ramírez, Sofía Gana, Soledad Garcés, Teresa Zúñiga, Ricardo Araya, Jorge Gaete

**Affiliations:** ^1^Faculty of Education, Research Center for Students Mental Health (ISME), Universidad de los Andes, Santiago, Chile; ^2^Agencia Nacional de Investigación y Desarrollo (ANID), Millennium Science Initiative Program, Millennium Nucleus to Improve the Mental Health of Adolescents and Youths, Imhay, Santiago, Chile; ^3^Fundación para la Convivencia Digital, Santiago, Chile; ^4^Department of Health Service and Population Research, King's College London, London, United Kingdom

**Keywords:** cellphone, videogame, academic performance, life satisfaction, children, adolescents

## Abstract

**Introduction:** In the last years, there has been a rise in the use of technology among children and adolescents, which has led to a greater concern about its impact on their socioemotional and cognitive development.

**Aims:** To explore the time spent using technology, the risk perception of its use by students, and the association between frequency of technology use and life satisfaction and academic performance among children and adolescents in Chile. Additionally, we explored the mediating effect of sleep deprivation on these outcomes.

**Methods:** This is a cross-sectional study, where 2,440 students (9-12 years old) from 13 schools participated. Data was collected using an online survey answered by students during school hours. The association analyses were performed using multivariable regression models considering life satisfaction and academic performance as dependent variables. Additionally, mediating analyses were conducted using structural equation modeling.

**Results:** Time watching television and using cellphones were similar on weekdays, and playing video games was the most frequent activity during weekends. A 42.1% of students reported playing online video games with strangers, and 12.7% had sleep deprivation. Lower self-reported academic performance was associated with cyberbullying victimization, sleep deprivation, being hacked, exposure to violent content, time spent using cell phones during weekdays and weekends, and playing video games during weekdays. Students who perceived that playing video games after 9 pm affected their sleep had a higher academic performance. There was a clear mediating effect of sleep deprivation in the relationship between time spent using a cellphone during weekdays and weekends and playing video games during weekdays and GPA.

**Conclusions:** Time spent using technological devices was not associated with life satisfaction; however, the time spent using cell phones and playing video games was related to lower self-reported academic performance, mediated by sleep deprivation. Future research may focus on a better understanding of the mechanisms involved in the effect of technology use on sleep routines among adolescents and potential interventions to reduce its impact on academic performance.

## Introduction

The use of new technologies such as smartphones, tablets, videogame consoles, and access to the Internet is more massive and starts at a younger age every day (1). This rise in use has led to a greater concern about the consequences of technology use and its impact on children and adolescents' emotional and cognitive development (1). For instance, a report in the UK showed that internet use of children and early adolescents between the ages of 5-15 increased from 9 h (2007) to 15 h (2016) per week (2). Furthermore, a recent report in the United States found that children and adolescents under the age of 12 had high engagement rates with technology devices, and 71% of the parents were at least somewhat concerned that their child spent too much time in front of screens (3). In Chile, a recent report (4) showed that 84.5% of households have an internet connection, and the preferred device by children and adolescents was smartphones (5). These figures are in pre-pandemic times. During the SARS-Cov-2 pandemic, there has been a significant increase in the use of electronics, mainly explained by the change of education from face-to-face interaction to remote learning. For example, a German study (6) reported a rise in video game use during the pandemic with a mean of 139 min spent daily playing video games on a weekday compared to about 79 min in September 2019 before the pandemic among 10-17 year-olds.

The evidence of the impact of technology use on the development of children and adolescents is still debatable (1, 7–9). On the one hand, adverse outcomes have been associated with technology use. A recent systematic review stated that technology use is associated with unfavorable psychological outcomes (7). A longitudinal study among adolescents showed that the higher the number of hours spent using technological devices, the lower life satisfaction (10). Additionally, exposure to screens has been related to poorer cognitive and academic functioning, including problems with attention (11), language (11), memory (12), learning (12), and visuospatial processing (7). Moreover, gaming addiction is a rising concern with a prevalence ranging from 2.0 to 3.1% among the young population worldwide (13). Recently the World Health Organization (WHO) included Gaming disorder in the 11th Revision of the International Classification of Diseases (ICD-11) (14), describing this disorder as a pattern of gaming behavior (“digital-gaming” or “video-gaming”) characterized by impaired control over gaming, that results in significant impairment in personal, family, or social functioning. This disorder has also been recently included in the Diagnostic and Statistical Manual of Mental Disorders (DSM-5) (15), specifically on the category Conditions for Further Study as Internet Gaming Disorder (IGD). In addition, another critical problem associated with technology use is the negative disruption of sleep patterns (1). For instance, exposure to the light of the devices in the evening and night, especially with an emotional investment in their use, has been associated with worse sleep quality and reduced sleep duration (16). Finally, there are other risks associated with Internet use. For example, online peer interaction increases the risk for cyberbullying (17, 18), which has a clear negative impact on mental health among victims, perpetrators, and even bystanders (19); furthermore, children and adolescents can access violent content, pornography, and communication with strangers, which in turn may put them at risk of suffering grooming (1).

On the other hand, studies have found that the use of technology may be beneficial. A recent study reported that adolescents who were light users of digital media had slightly higher wellbeing than non-users (20). Additionally, technological devices have facilitated learning processes, particularly for early mathematics learning (1). For instance, one systematic review found evidence of learning benefits of interactive apps among young children (21). One study found that among students who had below-average reading skills, higher Internet use was associated with improving those skills (22). Another study showed that playing computer games was associated with increased scores on comprehension and applied problems tests among children (23). Similarly, one study reported that if digital skills are developed during early childhood, a significantly positive association can be found in school performance later in life (24). Finally, technology use may help the promotion of social engagement with virtual friends and could develop relevant digital skills for the 21st-century labor market (1).

Including another dimension to the discussion of the effect of technology use, some new research cites a potential “Goldilocks effect” in terms of technology use (1). This effect suggests that a moderate engagement in online and digital activities might be beneficial in terms of wellbeing, whereas too much or too little might be detrimental (8, 9).

To understand the mechanisms associated with the effect of technology use, some studies have explored the pathway of the relationship between technology use and life satisfaction and academic performance. Specifically, studies have examined the mediation effect of sleep deprivation in this relationship. For instance, one study found that the time spent using electronic media decreased academic performance, and sleep time was a significant mediator (β = −0.88; *p*-value ≤ 0.05) (25). Another study using pathway analysis reported that the association between time spent using technological devices and attention problems was mediated by daily sleeping hours (β = −0.08; *p*-value ≤ 0.001) (26). Regarding life satisfaction, one study conducted in three European countries found that sleep duration partly mediated the association between computer use and well-being among 15 years old adolescents (27).

Chile is no exception to this knowledge gap. The only available study among children and adolescents that explored technology use, investigated the association of Internet use and life satisfaction, with no focus on academic performance nor mediation analysis (28).

The aims of this study were: (1) to describe the frequency of the time spent using technology; (2) the risk perception of its use by students; (3) the association between frequency of technology use and self-reported academic performance and life satisfaction among children and adolescents in Chile; and (4) the mediating effect of sleep deprivation on these associations.

## Materials and Methods

### Study Design, Procedure, Participants, and Ethical Considerations

This was a cross-sectional study. A total of 13 schools were invited to be part of the present study, and all of them agreed to participate. The 13 schools had a total of 2,765 eligible students. From this pool of students, a total of 2,491 parents or primary caregivers agreed to participate and gave written informed consent. Finally, 2,440 (88.2%) students gave written assent to participate. The sample size was 2,440 students attending from 4th to 7th grade (9–12 years old). The inclusion criteria of the participating schools were: (1) To have 4th–7th grades, and (2) To have a computer lab with internet access.

Data collection assessment took place in August-September 2019. The instrument used to collect data was an online questionnaire. This was a self-reported questionnaire answered by the students during school hours (A 45-min class was given to the research team), at the school computer labs, under the supervision of research assistants.

Regarding ethical considerations, data was collected following the Declaration of Helsinki, with the approval of the ethics committee of the Universidad de los Andes (CEC201948 August 7th, 2019). School authorities were informed about the study aims, methods, and assessments, and afterward, they agreed to participate. Then, an informed and written consent form was sent to the parents or primary caregivers to accept the participation of the students. Finally, informed and written assent was also provided by all students. The students and their parents or main caregivers did not receive any reimbursement for participating in this study. Confidentiality was granted during the whole assessment and the information was handled and coded by an independent statistician.

### Measures

#### Dependent Variables

(a)Self-reported academic performance: students reported their final grade point average (GPA) in the previous semester. In Chile, the marking scores go between 1 and 7, and 4 is the minimum score to approve the subject. We used this variable as continues.
(b)Life satisfaction: this outcome was measured using the Students' Life Satisfaction Scale (29). It has seven statements about the appreciation of life in general. Some of the statements are “I have a good life,” “I have what I want in life.” Students answered on a scale between 1 = “*totally disagree*” and 6 = “*totally agreed*.” The total score is the sum of the seven items (range: 7–42). Children and adolescents between 8 and 18 years old can answer this scale. It has been widely used and validated with good reliability and validity indicators (30). In Chile, there is a validated version of this scale showing good psychometric properties (31). The internal consistency in our sample was ω = 0.79.

#### Independent Variables

(a)Time spent using technological devices during weekdays. We explored the number of hours spent every weekday using cell phones and computers, playing video games, and watching TV. All questions were answered as 0 = *Never*; 0.5 = *half hour per day*; 1 = *1 h per day*: 2 = *2 h per day*; 3 = *3 h per day*; 4 = *4 h per day*; 5 = *5 h*\ *per day*; 6 = *6 h per day*; 7 = *7 or more hours per day*.
(b)Time spent using technological devices during weekends. We explored the number of hours spent every day of weekends using cell phones and computers, playing video games, and watching TV. All questions were answered as 0 = *Never*; 0.5 = *half hour per day*; 1 = *1 h per day*: 2 = *2 h per day*; 3 = *3 h per day*; 4 = *4 h per day*; 5 = *5 h per day*; 6 = *6 h per day*; 7 = *7 or more hours per day*.
(c)Digital technology and social media risk perception. Several items explored the risk perception of using technologies and risk exposure, such as receiving messages with violence or rude content.
(d)Online risk experiences. Students were asked if they have experienced the following events in the last 2 months: received unwanted violent content (e.g., photos, videos), had been hacked, exposed to violent content (e.g., photos, videos) on purpose, and played online games with strangers. All questions were answered as 0 = *No* or 1 = *Yes*.
(e)Video game dependence indicators and perception of negative effects related to video game use. Students were asked to agree (point score = 1) or disagree (point score = 0) with several statements. Two items assessed video games dependence: “Playing video games is an addiction to me and it is hard to stop” and “Playing video games calm me down.” The first statement can be considered closely related to the criterion “Unsuccessful attempts to control the participation in Internet games” of the diagnostic criteria of Internet Gaming Disorder (IGD). The second statement may be related to the following two criteria of IGD: (a) “Withdrawal symptoms when Internet gaming is taken away.” Withdrawal symptoms are typically described as irritability, anxiety, or sadness; and (b) “Use of Internet games to escape or relieve a negative mood (e.g., feelings of helplessness, guilt, anxiety)” (12). Additionally, as a perception of negative effects related to video game use, students were asked if they agreed with the statements “Playing video games makes me violent” and “Playing video games after 9 pm affects my sleep.”
(f)Cyberbullying: We asked about cyberbullying using three questions:
  a.Victimization: “Have I received cruel and hurtful messages, photos, or videos through the internet or social networks in the last 2 months?” (No = 0, Yes = 1)
  b.Perpetration: “Have I sent cruel and hurtful messages or offensive photographs through the internet or social networks in the last 2 months?” (No = 0, Yes = 1)
  c.Bystanders: “Have I seen others sending cruel and hurtful messages or offensive photographs through the internet or social networks in the last 2 months?” (No = 0, Yes = 1).
(g)Sleep deprivation: We asked children the time they go to bed and the time they wake up. We calculated the number of hours of sleep per night. Later, we categorized the sleep time into two groups, according to the recommended hours of sleep for these age groups: minimum of 8 h and a maximum of 12 h (32). The two new groups were: sleep deprivation <8 h and normal sleep ≥ 8 h. No students reported more than 12 h of sleep.

### Statistical Analyses

A descriptive analysis was performed, calculating the mean age by class year, weekly hours dedicated to technology by grade, life satisfaction, and GPA. Then, to represent the relative frequencies, percentages were calculated, with 95% confidence intervals, for sociodemographic variables, risky online experiences, perception of indicators of dependence on video games, and adverse effects of video games, cyberbullying, and sleep deprivation.

Unadjusted regression models were performed using all measured risk factor variables as independent variables and life satisfaction and GPA as dependent variables. Outcomes were analyzed as continuous variables. The coefficients were examined with 95% confidence intervals, and the cutoff for statistical significance was established with a *p*-value < 0.05. All significant variables in the unadjusted analyses were included in a multivariable regression modeling along with age and gender. Age and gender are included in all full models regardless of the strength of the association reached in the univariable models because they are considered important confounding variables. The cutoff for statistical significance was established with a *p*-value < 0.05.

Finally, if any of the variables of “time spent” using a cellphone or computer, watching TV, and playing video games were significantly associated with any of the outcomes, they entered in the mediation modeling. Additionally, if sleep deprivation was associated with any of the outcomes in the multivariable analyses, this variable was included in the mediation modeling. We explored the mediation role of sleep deprivation in the association between “time spent” using technological devices and life satisfaction or self-reported academic performance. The mediating analyses were performed using structural equation modeling, adjusted by age and gender. The cutoff for statistical significance was established with a *p*-value < 0.05. All statistical analyses were performed using Stata 15.

## Results

### Descriptive Features

A total of 2,440 students participated in the study. A 54% of students were female, and their ages ranged between 9 and 12. The average age for 4th grade was 9.4 years old, for 5th grade was 10, for 6th grade was 11.3, and for 7th grade was 12.3. Most of the students (77.4%) lived with both parents. The mean score of the Life Satisfaction scale was 30 (SD = 0.2) and for self-reported GPA was 6.2 (SD = 0.5) (see Table 1).

**Table 1:** Demographic features, life satisfaction and self-reported academic performance (GPA).

**Variables**	***n***	**%/mean**	**[95% CI]/(SD)**
Gender
Female	1,317	54.0	[52.0–55.9]
Male	1,123	46.0	[44.1–48.0]
Class grade
4th	765	31.4	[29.5–33.2]
5th	601	24.6	[23.0–26.4]
6th	532	21.8	[20.2–23.5]
7th	542	22.2	[20.6–23.9]
Age by class grade
4th	765	9.4	(0.6)
5th	601	10.3	(0.5)
6th	532	11.3	(0.5)
7th	542	12.3	(0.5)
Total	2,440	10.6	(1.2)
Living with both parents	1,888	77.4	[75.7–79.0]
Life satisfaction (7–42)	2,056	30.0	(0.2)
GPA (1–7)	2,379	6.2	(0.5)

*n, number of participants; CI, Confidence interval; SD, Standard deviation*.

### Time Spent Using Technological Devices During Weekdays

Students spent between 1.56 (4th graders) and 1.01 (7th graders) hours watching TV. The time spent playing video games was between 1.31 (4th graders) and 1.14 (7th graders). In both cases, the time spent using these technological devices was descending as the grade increased. On the other hand, using a computer and cellphone, the time spent on those devices increases with grade. The time spent using a computer was between 0.41 (4th graders) and 0.60 (7th graders) hours, and for the cellphone was between 0.89 (4th graders) and 2.03 (7th graders) hours. Regarding the distribution of the time spent using technological devices, most students use these devices between 0.5 to 2 h. A 17% of students reported using cellphones over 2 h a day (see Tables 2A,B).

**Table 2A:** Average hours spent using technological devices every weekday by grade (*n* = 2,309; Missing data = 131).

	**Television**		**Computer**		**Cellphone**		**Video games**	
**Grade**	***n***	**Mean (SD)**	***N***	**Mean (SD)**	***n***	**Mean (SD)**	***n***	**Mean (SD)**
4th	704	1.56 (1.6)	704	0.41 (0.9)	704	0.89 (1.5)	704	1.31 (1.7)
5th	573	1.42 (1.5)	573	0.51 (1.0)	573	1.19 (1.6)	573	1.25 (1.7)
6th	504	1.16 (1.2)	504	0.56 (1.1)	504	1.39 (1.7)	504	1.22 (1.6)
7th	528	1.01 (1.3)	528	0.60 (1.0)	528	2.03 (2.0)	528	1.14 (1.4)
Total	2,309	1.33 (1.4)	2,309	0.51 (1.0)	2,309	1.33 (1.7)	2,309	1.23 (1.6)

“*Total” includes “0 = Never” answers; n, number of participants; SD, Standard deviation*.

**Table 2B:** Percentage of students by time spent using technological devices during weekdays (*n* = 2,309; Missing data = 131).

	**Television**		**Computer**		**Cellphone**		**Video games**	
**Time**	***n***	**% [95% CI]**	***n***	**% [95% CI]**	***n***	**% [95% CI]**	***n***	**% [95% CI]**
Never	273	11.2 [10.0–12.5]	1,243	50.9 [49.0–52.9]	648	26.6 [24.8–28.3]	683	28.0 [26.2–29.8]
0.5 h	782	32.1 [30.2–33.9]	644	26.4 [24.7–28.2]	601	24.6 [23.0–26.4]	557	22.8 [21.2–24.5]
1 h	563	23.1 [21.4–24.8]	220	9.0 [7.9–10.2]	398	16.3 [14.9–17.8]	422	17.3 [15.8–18.8]
2 h	358	14.7 [13.3–16.1]	105	4.3 [3.6–5.2]	248	10.2 [9.0–11.4]	304	12.5 [11.2–13.8]
3 h	145	5.9 [5.1–7.0]	41	1.7 [1.2–2.3]	158	6.5 [5.6–7.5]	128	5.3 [4.4–6.2]
4 h	83	3.4 [2.8–4.2]	24	1.0 [0.7–1.5]	80	3.3 [2.6–4.1]	79	3.2 [2.6–4.0]
5 h	38	1.6 [1.1–2.1]	7	0.3 [0.1–0.6]	52	2.1 [1.6–2.8]	41	1.7 [1.2–2.3]
6 h	24	1.0 [0.7–1.5]	5	0.2 [0.1–0.5]	34	1.4 [1.0–1.9]	27	1.1 [0.8–1.6]
7+ h	43	1.8 [1.3–2.4]	20	0.8 [0.5–1.3]	90	3.7 [3.0–4.5]	68	2.8 [2.2–3.5]

*n, number of participants; CI, Confidence Interval*.

### Time Spent Using Technological Devices During Weekends

Students spent between 1.76 (4th graders) and 1.36 (7th graders) hours watching TV. Moreover, this time was descending as the grade increased. On the other hand, using a computer and cellphone or playing video games, the time spent on those devices increases with grade. The time spent using a computer was between 0.51 (4th graders) and 0.74 (7th graders) hours, for the cellphone was between 1.01 (4th graders) and 2.45 (7th graders) hours, and for playing video games was between 1.73 (4th graders) and 1.88 (7th graders) hours. Regarding the distribution of the time spent using technological devices, most students use these devices between 0.5 and 2 h. A 28.2% of students reported playing videogames over 2 h a day on weekends (see Tables 3A,B).

**Table 3A:** Average hours spent using technological devices every day of weekends by grade (*n* = 2,309; Missing data = 131).

	**Television**		**Computer**		**Cellphone**		**Video games**	
**Grade**	***N***	**Mean (SD)**	***n***	**Mean (SD)**	***n***	**Mean (SD)**	***n***	**Mean (SD)**
4th	704	1.76 (1.8)	704	0.51 (1.1)	704	1.01 (1.7)	704	1.73 (2.0)
5th	573	1.70 (1.7)	573	0.61 (1.2)	573	1.38 (1.8)	573	1.84 (2.1)
6th	504	1.52 (1.6)	504	0.66 (1.3)	504	1.78 (2.0)	504	2.00 (2.1)
7th	528	1.36 (1.5)	528	0.74 (1.3)	528	2.45 (2.1)	528	1.88 (2.0)
Total	2,309	1.60 (1.7)	2,309	0.62 (1.2)	2,309	1.60 (1.9)	2,309	1.85 (2.0)

“*Total” includes “0 = Never” answers; n, number of participants; SD, Standard deviation*.

**Table 3B:** Percentage of students by time spent using technological devices every day of weekends (*n* = 2,309; Missing data = 131).

	**Television**		**Computer**		**Cellphone**		**Video games**	
**Time**	***n***	**% [95% CI]**	***n***	**% [95% CI]**	***n***	**% [95% CI]**	***n***	**% [95% CI]**
Never	278	11.4 [10.2–12.7]	1,214	52.6 [50.5–54.6]	564	24.4 [22.7–26.2]	512	22.2 [20.5–23.9]
0.5 h	646	26.5 [24.8–28.3]	585	25.3 [23.6–27.2]	584	25.3 [23.6–27.1]	453	19.6 [18.0–21.3]
1 h	522	21.4 [19.8–23.1]	246	10.7 [9.5–12.0]	351	15.2 [13.8–16.7]	360	15.6 [14.2–17.1]
2 h	384	15.7 [14.3–17.2]	125	5.4 [4.6–6.4]	281	12.2 [10.9–13.6]	333	14.4 [13.0–15.9]
3 h	215	8.8 [7.7–10.0]	49	2.1 [1.6–2.8]	166	7.2 [6.2–8.3]	230	10.0 [8.8–11.3]
4 h	88	3.6 [2.9–4.4]	35	1.5 [1.1–2.1]	117	5.1 [4.2–6.0]	127	5.5 [4.6–6.5]
5 h	50	2.1 [1.6–2.7]	11	0.5 [0.3–0.9]	75	3.3 [2.6–4.1]	92	4.0 [3.3–4.9]
6 h	42	1.7 [1.3–2.3]	9	0.4 [0.2–0.9]	48	2.1 [1.6–2.7]	51	2.2 [1.7–2.9]
7+ h	84	3.4 [2.8–4.2]	35	1.5 [1.1–2.1]	123	5.3 [4.5–6.3]	151	6.5 [5.6–7.6]

*n, number of participants; CI, Confidence Interval*.

### Online Risk Experiences

The most frequent experience was playing online games with strangers (42.1%). One out of three 4th graders have had this experience, and more than 50% of 7th graders have played with strangers. This experience was followed by “seen violent content online on purpose” (20.6%), especially among 7th graders (29.1%). The percentage of students who have received unwanted violent content was 12.9%, rising to 15.8% among 7th graders. Finally, 10% of the students declared that they had been hacked (see Table 4).

**Table 4:** Online risk experiences by grade (*n* = 2,198; Missing data = 242).

	**Had received unwanted violent content**		**Had been hacked**		**Had seen violent content on purpose**		**Had played online with strangers**	
**Grade**	***n***	**% [95% CI]**	***n***	**% [95% CI]**	***n***	**% [95% CI]**	***n***	**% [95% CI]**
4th	87	13.1 [10.7–15.8]	67	10.1 [8.0–12.6]	124	18.6 [15.8–21.8]	228	34.2 [30.7–37.9]
5th	56	10.4 [8.1–13.3]	51	9.5 [7.3–12.3]	97	18.1 [15.0–21.6]	222	41.3 [37.2–45.6]
6th	59	12.2 [9.6–15.5]	51	10.6 [8.1–13.6]	82	17.0 [13.9–20.6]	206	42.7 [38.3–47.1]
7th	81	15.8 [12.9–19.2]	51	10.0 [7.6–12.9]	149	29.1 [25.3–33.2]	270	52.7 [48.4–57.0]
Total	283	12.9 [11.5–14.3]	220	10.0 [8.8–11.3]	452	20.6 [18.9–22.3]	926	42.1 [40.1–44.2]

*n, number of participants; CI, Confidence interval*.

### Indicators of Video Game Dependence and Perception of Negative Effects

More than 50% of all students considered that they might have a video game addiction, and this opinion was reported in 63.4% of 4th graders. One out of four students declared that video games calm them down, and this percentage was very similar in all grades. Additionally, 35.2% of the students reported that video games make them violent, and the highest percentage was found among 4th graders (40.3%). Finally, more than 70% of them, in all grades, seem to know that playing video games after 9 pm deteriorates their sleep (see Table 5).

**Table 5:** Perception of indicators of dependence on video games and negative effects of video games (*n* = 1,630; Missing data = 827).

	**Video game dependence**				**Video game negative effects**			
	**VG are an addiction to me**		**VG calm me down**		**VG make me violent**		**Agree playing VG after 9 pm affects my sleep**	
**Grade**	***n***	**% [95% CI]**	***n***	**% [95% CI]**	***n***	**% [95% CI]**	***n***	**% [95% CI]**
4th	313	63.4 [59.0–67.5]	127	25.7 [22.0–29.8]	199	40.3 [36.0–44.7]	352	71.3 [67.1–75.1]
5th	220	57.3 [52.3–62.2]	96	25.0 [20.9–29.6]	120	31.3 [26.8–36.1]	276	71.9 [67.2–76.2]
6th	193	55.1 [50.0–60.3]	88	25.1 [20.9–30.0]	122	34.9 [30.0–40.0]	267	76.3 [71.5-80.5]
7th	200	51.9 [46.9–56.9]	98	25.5 [21.3–30.1]	126	32.7 [28.2–37.6]	274	71.2 [66.4–75.5]
Total	926	57.4 [55.0–60.0]	409	25.4 [23.3–27.5]	567	35.2 [32.9–37.5]	1169	72.5 [70.2–74.6]

*VG, Video games; n, number of participants; CI, Confidence interval*.

### Cyberbullying

Victimization was reported by 9.7% of students (ranging from 7.6%, among 5th graders, to 11.1% among 7th graders). Perpetration was reported by 4.4% of students, with the highest proportion among 6th graders (6.2%). One out of three students declared that they have witnessed others send offensive and hurtful messages or offensive photographs through the Internet or social networks (see Table 6).

**Table 6:** Cyberbullying by grade (*n* = 2,198; Missing data = 242).

	**Victimization**		**Perpetration**		**Bystanders**	
**Grade**	***n***	**% [95% CI]**	***n***	**% [95% CI]**	***n***	**% [95% CI]**
4th	70	10.5 [8.4–13.1]	30	4.5 [3.2–6.4]	187	28.1 [24.8–31.6]
5th	41	7.6 [5.7–10.2]	18	3.4 [2.1–5.3]	147	27.4 [23.8–31.3]
6th	45	9.3 [7.0–12.3]	30	6.2 [4.4–8.7]	168	34.8 [30.7–39.1]
7th	57	11.1 [8.7–14.2]	18	3.5 [2.2–5.5]	189	36.9 [32.8–41.2]
Total	213	9.7 [8.5–11.0]	96	4.4 [3.6–5.3]	691	31.4 [29.5–33.4]

*n, number of participants; CI, Confidence interval*.

### Sleep Deprivation

The percentage of students who slept < 8 h was 12.7%, ranging from 8.5% in 4th grade to 23.5% among 7th graders (see Table 7).

**Table 7:** Sleep deprivation by grade (*n* = 2,309; Missing data = 131).

	** < 8 h**		**≥8 h**	
**Grade**	***n***	**% [95% CI]**	**n**	**% [95% CI]**
4^th^	60	8.5 [6.7–10.8]	644	91.5 [89.2–93.3]
5^th^	52	9.1 [7.0–11.7]	521	90.9 [88.3–93.0]
6^th^	58	11.5 [9.0–14.6]	446	88.5 [85.4–91.0]
7th	124	23.5 [20.1-27.3]	404	76.5 [72.7–79.9]
Total	294	12.7 [11.4–14.2]	2015	87.3 [85.8–88.6]

*n, number of participants; CI, Confidence interval*.

### Associations

The model that included “Time spent using technological devices during weekdays” showed that time spent using a cellphone and playing video games was negatively associated with self-reported academic performance. Several online risk experiences were also associated with lower academic performance, such as playing with strangers and seeing violent content. Those students who believed that playing video games after 9 pm affects their sleep had a higher likelihood of higher GPA scores. Victimization was negatively associated with GPA. Finally, those students who slept fewer than 8 h had a higher risk of lower GPA.

The time spent using technological devices was not associated with lower scores on the life satisfaction scale. Among the other explored variables, only “Received unwanted violent content,” the opinion that playing video games “Make them violent,” and being a victim of cyberbullying were associated with lower life satisfaction (see Table 8).

**Table 8:** Associations between time spent using technological devices during weekdays and academic performance and life satisfaction.

	**Academic performance**						**Life satisfaction**					
	**Unadjusted**			**Adjusted**			**Unadjusted**			**Adjusted**		
**Variables**	**Beta**	**[95% CI]**	***P*-value**	**Beta**	**[95% CI]**	***P*-value**	**Beta**	**[95% CI]**	***P*-value**	**Beta**	**[95% CI]**	***P*-value**
**Time spent weekdays**												
Television	−0.02	[−0.04 to −0.01]	**0.002**	−0.16	[−0.35 to 0.00]	0.089	−0.14	[−0.37 to 0.10]	0.253	N/A	N/A	N/A
Computer	−0.04	[−0.06 to −0.02]	**0.000**	0.16	[−0.01 to 0.04]	0.253	−0.43	[−0.76 to −0.10]	**0.011**	−0.16	[−0.56 to 0.24]	0.429
Cellphone	−0.05	[−0.06 to −0.04]	**0.000**	−0.03	[−0.04 to −0.01]	**0.002**	−0.30	[−0.48 to −0.11]	**0.002**	0.07	[−0.18 to 0.32]	0.575
Video game	−0.05	[−0.07 to −0.04]	**0.000**	−0.03	[−0.04 to −0.01]	**0.002**	−0.24	[−0.45 to −0.04]	**0.022**	−0.08	[−0.34 to 0.18]	0.536
**Risk experiences**												
Had received unwanted violent content	−0.15	[−0.21 to −0.08]	**0.000**	−0.04	[−0.12 to 0.04]	0.316	−2.76	[−3.74 to −1.78]	**0.000**	−1.43	[−2.6 to −0.26]	**0.016**
Had been hacked	−0.22	[−0.29 to −0.15]	**0.000**	−0.15	[−0.23 to −0.07]	**0.000**	−1.41	[−2.5 to −0.30]	**0.013**	0.38	[−0.86 to 1.62]	0.550
Had seen violent content on purpose	−0.17	[−0.22 to −0.11]	**0.000**	−0.07	[−0.13 to −0.00]	**0.049**	−1.86	[−2.67 to −1.04]	**0.000**	−0.35	[−1.37 to 0.66]	0.494
Had played online with strangers	−0.11	[−0.15 to −0.06]	**0.000**	−0.06	[0.00 to 0.11]	**0.045**	−0.80	[−1.47 to −0.13]	**0.020**	−0.56	[−1.39 to 0.27]	0.187
**Video game dependence and**												
**negative effects**												
Calm them down	0.01	[−0.05 to 0.07]	0.721	N/A	N/A	N/A	0.08	[−0.82 to 0.98]	0.863	N/A	N/A	N/A
Are an addiction	−0.03	[−0.08 to 0.03]	0.335	N/A	N/A	N/A	−0.90	[−1.69 to −0.12]	**0.024**	−0.48	[−1.30 to 0.34]	0.248
Make them violent	0.01	[−0.05 to 0.06]	0.764	N/A	N/A	N/A	−1.34	[−2.15 to −0.53]	**0.001**	−0.95	[−1.78 to −0.12]	**0.025**
Agree playing late affects their sleep	0.10	[0.04 to 0.16]	**0.001**	0.08	[0.03 to 0.14]	**0.004**	0.28	[−0.59 to 1.15]	0.531	N/A	N/A	N/A
**Cyberbullying**												
Victim	−0.18	[−0.25 to −0.10]	**0.000**	−0.09	[−0.18 to −0.00]	**0.043**	−4.02	[−5.14 to −2.91]	**0.000**	−3.44	[−4.81 to −2.08]	**0.000**
Perpetrator	−0.23	[−0.34 to −0.12]	**0.000**	−0.09	[−0.21 to 0.02]	0.117	−1.83	[−3.5 to −0.16]	**0.032**	−0.63	[−2.46 to 1.20]	0.501
Bystander	−0.09	[−0.14 to −0.04]	**0.000**	−0.01	[−0.07 to 0.04]	0.697	−0.85	[−1.56 to −0.13]	**0.020**	0.01	[−0.85 to 0.86]	0.986
**Sleep routine**												
Sleep deprivation	−0.21	[−0.27 to −0.14]	**0.000**	−0.08	[−0.16 to −0.01]	**0.031**	−2.16	[−3.14 to −1.17]	**0.000**	−0.91	[−2.03 to 0.22]	0.115

*The number of observations for academic performance outcome is 1,566. The number of observations for life satisfaction outcome is 1,495. P-values ≤ 0.05 are in bold; N/A, Not applicable; CI, Confidence interval*.

On the other hand, the model that included “Time spent using technological devices during weekends” showed that time spent using a cellphone was negatively associated with self-reported academic performance. Similar to the results from the weekdays model, several online risk experiences were associated with lower academic performance, except for playing online with strangers. Finally, those students who slept fewer than 8 hours had a higher risk of lower GPA.

Similarly to weekdays, the time spent using technological devices was not associated with lower scores on the life satisfaction scale. Among the other explored variables, “Received unwanted violent content,” the opinion that playing video games “Make them violent,” and cyberbullying victimization were associated with lower life satisfaction (see Table 9).

**Table 9:** Associations between time spent using technological devices during weekends and academic performance and life satisfaction.

	**Academic performance**						**Life satisfaction**					
	**Unadjusted**			**Adjusted**			**Unadjusted**			**Adjusted**		
**Variables**	**Beta**	**[95% CI]**	***P*-value**	**Beta**	**[95% CI]**	***P*-value**	**Beta**	**[95% CI]**	***P*-value**	**Beta**	**[95% CI]**	***P*-value**
**Time spent weekends**												
Television	−0.01	[−0.02 to 0.00]	0.190	N/A	N/A	N/A	−0.12	[−0.32 to 0.08]	0.238	N/A	N/A	N/A
Computer	−0.03	[−0.05 to −0.02]	**0.000**	0.00	[−0.02 to 0.02]	0.764	−0.30	[−0.58 to −0.03]	**0.030**	−0.16	[−0.47 to 0.16]	0.330
Cellphone	−0.05	[−0.06 to −0.04]	**0.000**	−0.03	[−0.05 to −0.02]	**0.000**	−0.24	[−0.41 to 0.74]	**0.005**	0.03	[−0.19 to 0.25]	0.771
Video game	−0.04	[−0.05 to −0.03]	**0.000**	−0.00	[−0.02 to 0.01]	0.747	0.01	[−0.15 to 0.17]	0.903	N/A	N/A	N/A
**Risk experiences**												
Had received unwanted violent content	−0.15	[−0.21 to −0.08]	**0.000**	−0.03	[−0.11 to 0.04]	0.407	−2.76	[−3.74 to −1.78]	**0.000**	−1.43	[−2.6 to −0.25]	**0.017**
Had been hacked	−0.22	[−0.29 to −0.15]	**0.000**	−0.15	[−0.23 to −0.07]	**0.000**	−1.41	[−2.5 to −0.30]	**0.013**	0.39	[−0.85 to 1.63]	0.535
Had seen violent content on purpose	−0.17	[−0.22 to −0.11]	**0.000**	−0.07	[−0.13 to −0.01]	**0.029**	−1.86	[−2.67 to −1.04]	**0.000**	−0.38	[−1.38 to 0.63]	0.464
Had played with strangers	−0.11	[−0.15 to −0.06]	**0.000**	0.05	[0.00 to 0.11]	0.058	−0.80	[−1.47 to −0.13]	**0.020**	−0.59	[−1.41 to 0.24]	0.165
**Video game dependence and**												
**negative effects**												
Calm them down	0.01	[−0.05 to 0.07]	0.721	N/A	N/A	N/A	0.08	[−0.82 to 0.98]	0.863	N/A	N/A	N/A
Are an addiction	−0.03	[−0.08 to 0.03]	0.335	N/A	N/A	N/A	−0.90	[−1.69 to −0.12]	**0.024**	−0.50	[−1.32 to 0.31]	0.225
Make them violent	0.01	[−0.05 to 0.06]	0.764	N/A	N/A	N/A	−1.34	[−2.15 to −0.53]	**0.001**	−0.96	[−1.80 to −0.13]	**0.023**
Agree playing late affects their sleep	0.10	[0.04 to 0.16]	**0.001**	0.08	[0.02 to 0.13]	**0.008**	0.28	[−0.59 to 1.15]	0.531	N/A	N/A	N/A
**Cyberbullying**												
Victim	−0.18	[−0.25 to −0.10]	**0.000**	−0.10	[−0.19 to −0.01]	**0.024**	−4.02	[−5.14 to −2.91]	**0.000**	−3.47	[−4.83 to −2.12]	**0.000**
Perpetrator	−0.23	[−0.34 to −0.12]	**0.000**	−0.10	[−0.21 to 0.02]	0.108	−1.83	[−3.5 to −0.16]	**0.032**	−0.62	[−2.44 to 1.21]	0.507
Bystander	−0.09	[−0.14 to −0.04]	**0.000**	−0.01	[−0.06 to 0.05]	0.849	−0.85	[−1.56 to −0.13]	**0.020**	0.01	[−0.84 to 0.86]	0.980
**Sleep routine**												
Sleep deprivation	−0.21	[−0.27 to −0.14]	**0.000**	−0.09	[−0.17 to −0.02]	**0.013**	−2.16	[−3.14 to −1.17]	**0.000**	−0.94	[−2.06 to 0.18]	0.101

*The number of observations for academic performance outcome is 1,566. The number of observations for life satisfaction outcome is 1,495. P-values ≤ 0.05 are in bold; N/A, Not applicable; CI, Confidence interval*.

### Mediation Modeling Analyses

**Figure 1:**
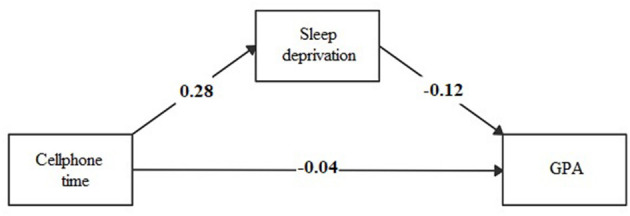
Mediation model of sleep deprivation in the association between time spent during weekdays using cellphone and academic performance (GPA).

**Figure 2:**
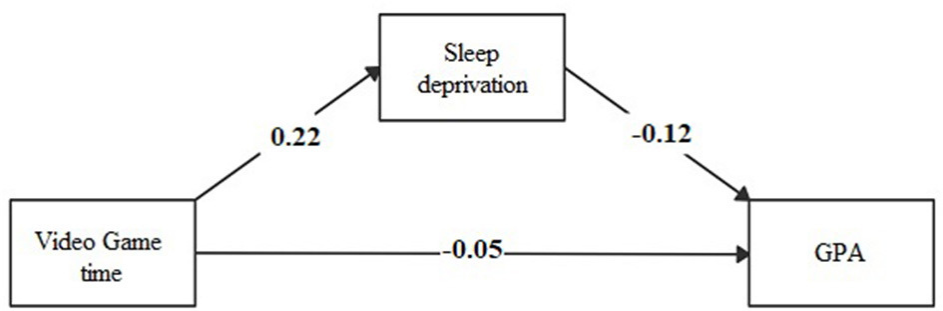
Mediation model of sleep deprivation in the association between time spent during weekdays playing video games and academic performance (GPA).

**Figure 3:**
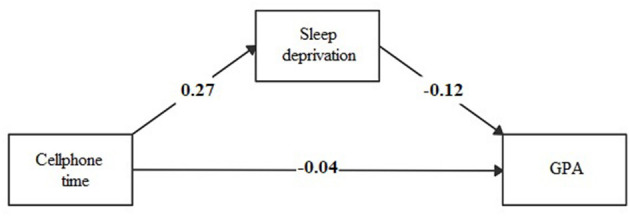
Mediation model of sleep deprivation in the association between time spent during weekends using cellphone and academic performance (GPA).

The association between time spent using a cellphone during the weekdays or weekends and playing video games during the weekdays directly affected GPA and an indirect effect mediated by sleep deprivation (see Figures 1–3; Supplementary Tables).

## Discussion

Technological devices are widely used among early adolescents. The main risks associated with its use were playing video games with strangers, recognizing a potential video game addiction, and 72.5% of students agreed that playing video games after 9 pm affects their sleep. Time using cellphones during weekdays or weekends and playing video games during weekdays were associated with poorer academic performance, while no associations were found between time spent using any technological devices and life satisfaction. Furthermore, our research is one of the few studies exploring and finding a mediation effect of sleep deprivation in the association of technology use and academic performance among children and early adolescents.

Life satisfaction was lower among those students who received unwanted violent content, those who agreed that video games could make them violent, and those who were victims of cyberbullying. These associations have also been found elsewhere (33). However, life satisfaction was not associated with any of the variables exploring time spent using technological devices. Our finding is consistent with the current view of this association, saying that, at a population level, technology use has a negligible effect on wellbeing (34). For instance, results from the analysis of three large-scale social datasets showed that the association between digital technology use and adolescent wellbeing was negative but small, explaining at most 0.4% of the variation in wellbeing (34). Another consideration of this association comes from studies showing that some use of technology may be beneficial. For example, a large survey of English adolescents, using the Warwick-Edinburgh Mental Well-Being Scale, showed that moderate engagement in online and digital activities might be beneficial in terms of wellbeing, and too little, might be detrimental, supporting the Goldilocks' hypothesis (9). In addition, the problem may not be the technology use itself but the heavy use of digital devices. The heavy use may displace time that might otherwise be spent in in-person social interaction, an activity with clear and established links to better mental health and happiness (35). Finally, other potential reasons explaining the elsewhere mixed results of the association between technology used and wellbeing may be the culturally-based differences between countries of adolescents' perceptions of wellbeing or life satisfaction (36) and the scarce research ruling out potential confounders with a comprehensive approach (37) as the one we conducted in this study.

Our results highlighted the effect of using a cellphone or playing video games on academic performance. Most of the literature agrees with our results. For example, in one longitudinal study conducted in Germany (38), lower use of the computer or Internet resulted in better school performance in mathematics. Another large study conducted in the United States also found that screen time was related to decreased academic performance (39).

Other variables were also related to poor academic performance, such as cyberbullying victimization, being hacked, and sleep deprivation. On the contrary, students who perceived a risk of playing video games after 9 pm had higher school achievements. The latter result seems consistent with one study in Spain, where adolescents with the highest academic performance spent more time sleeping and less time using screen media (40). High achievement students seem to understand better the importance of having a good duration and quality of sleeping to have good grades (41). Being mindful of the risks of playing video games at night should be included in the work with parents when implementing interventions to promote responsible use of screen time.

The effect of the time spent using technological devices on academic performance may have several explanations. We explored the mediating effect of sleep deprivation in this association. The time spent using a cellphone during weekdays and playing video games during weekends had an indirect effect of sleep deprivation on GPA, mediated by sleep deprivation. The magnitude of the indirect effect of sleep deprivation was three-folds of the direct effect between the time spent using a cellphone and GPA, and double the direct effect between the time spent playing video games and GPA. To our knowledge, few studies are exploring the role of sleep deprivation as a mediator of the association between technology use and academic performance among children or adolescents. In one study conducted in England (25), electronic media use before bedtime decreased academic performance via the mediating pathway of sleep time (β = −0.88; *p*-value ≤ 0.05). Another study conducted in the United States (26) reported that the association between time spent using the computer, playing video games, and watching television reduced the attention in class, according to teachers' report, mediated by daily sleeping hours (β = −0.08; *p*-value ≤ 0.001). In addition, a study conducted in Taiwan (42) found that disturbed sleep due to social media had an indirect effect (β = −0.07; *p*-value ≤ 0.05) on school performance. It is known that the blue light produced by screens alters the production of melatonin and, therefore, the rhythm of sleep, which in turn affects cognition (43, 44).

The findings of this study should be widely disseminated to the community to increase awareness of the effect of excessive use of technological devices, especially on sleep deprivation, and help parents mediate the use of technology and support strategies to reduce screen time, especially during evenings and nights, when blue light of devices may alter adolescents' sleep routines (1). Additionally, policy-makers should provide recommendations and funding to implement evidence-based interventions to promote responsible use of technological devices among children and adolescents.

This study has some limitations. First, this is a cross-sectional study, so no causality can be concluded regarding the associations. Second, this was a self-reported questionnaire, so social desirability, and information bias may exist. Answers provided by the students may have overestimated or underestimated the use of technology. Additionally, the questions about video game dependence and perception of negative effects were not part of validated instruments, therefore these results should be interpreted carefully. For example, in the statement “Playing video games is an addiction to me and it is hard to stop,” it is difficult to be sure if students fully understood the concept of addiction. Fourth, the questions regarding technology use did not specify if its use was restricted to leisure time. Also, we did not ask for details about what students were doing while using technological devices. Regarding the first point, schools in Chile, when the research was conducted, had not permitted the use of cellphones, playing videogames, or watching TV at schools, an allowed using computers to perform academic work only at computer labs. So, it is most probably that students used cellphones and computers, watched TV, and played videogames after school hours. Regarding the content consumed by students while using technological devices, we only have data of the risks experienced by students (Table 4). Still, we do not have detailed information on all the content consumed, which was beyond the aims of our study. Fifth, we did not ask in detail if the use of computers or internet access was in the school classroom, computer labs, or at home. Using computers in classrooms or at computer labs was not a common practice during data collection. And indeed, many students did not have computers or internet access at home. This may explain why some students reported that they have never used computers. Sixth, analysis was restricted to young adolescents; therefore, the results might not apply to older age groups. Seventh, potentially important truly contextual variables such as socioeconomic status and mental health problems were unavailable. Eighth, our study may have introduced some selection bias because the inclusion criteria stated that the schools need computer labs to participate. Therefore, schools less affluent may be underrepresented. Among the strengths of our study, we included a large sample size (N = 2,440). We performed sophisticated data analyses with structural equation modeling and included different explicatory variables for the outcomes.

Our results highlight the mediation role of sleep deprivation on the association pathway between technology use and academic performance. If used with moderation and respecting sleep hours, technology use may not be detrimental, but this hypothesis needs further research. Additionally, future investigations may focus on other mechanisms involved in the relationship between using technological devices and academic performance or life satisfaction.

## Data Availability Statement

The raw data supporting the conclusions of this article will be made available by the authors, without undue reservation.

## Ethics Statement

The studies involving human participants were reviewed and approved by Ethical and Scientific Committee of the Universidad de los Andes, Santiago, Chile. (CEC201948 August 7th, 2019). Written informed consent to participate in this study was provided by the participants' legal guardian/next of kin.

## Author Contributions

SR: software, formal analysis, and writing—original draft. SGan: writing—review and editing. SGar: resources. TZ: resources. RA: writing—review and editing. JG: conceptualization, methodology, validation, formal analysis, investigation, data curation, writing—review and editing, visualization, supervision, project administration, and funding acquisition. All authors contributed to the article and approved the submitted version.

## Funding

This research was funded by Fundación para la Convivencia Digital and ANID—Millennium Science Initiative Program—NCS17_035.

## Conflict of Interest

The authors declare that the research was conducted in the absence of any commercial or financial relationships that could be construed as a potential conflict of interest.

## Publisher's Note

All claims expressed in this article are solely those of the authors and do not necessarily represent those of their affiliated organizations, or those of the publisher, the editors and the reviewers. Any product that may be evaluated in this article, or claim that may be made by its manufacturer, is not guaranteed or endorsed by the publisher.

## Abbreviations

ICD-11: International Classification of Diseases
DSM-5: Diagnostic and Statistical Manual of Mental Disorders
IGD: Internet Gaming Disorder
GPA: Grade Point Average.
