# Supplementary material for: Use of Technology and Its Association With Academic Performance and Life Satisfaction Among Children and Adolescents
Source: Front Psychiatry. 2021 Nov 11;12:764054. doi: 10.3389/fpsyt.2021.764054 (PMC8631867; doi:10.3389/fpsyt.2021.764054)
Supplement: Supplementary file 1 [file Table_1.DOCX]

Supplementary Tables

Table a: Mediation analysis of sleep deprivation in the association between cellphone time during weekdays and Academic performance (GPA), using structural equation model.

| GPA (outcome) | Beta | [95% CI] | P value |
| --- | --- | --- | --- |
| Sleep deprivation | -0.12 | [-0.19- -0.06] | 0.000 |
| Age | -0.07 | [-0.09- -0.06] | 0.000 |
| Gender | 0.12 | [0.08- 0.17] | 0.000 |
| Cellphone time | -0.04 | [-0.05- -0.03] | 0.000 |
| Sleep deprivation over device |  |  |  |
| Cellphone time | 0.28 | [0.22- 0.34] | 0.000 |

Note: Model explored cellphone time effect on GPA mediated by sleep deprivation and adjusted with age and gender variables; CI = Confidence interval.

Table b: Mediation analysis of sleep deprivation in the association between video game time during weekdays and Academic performance (GPA), using structural equation model.

| GPA (outcome) | Beta | [95% CI] | P value |
| --- | --- | --- | --- |
| Sleep deprivation | -0.12 | [-0.18- -0.06] | 0.000 |
| Age | -0.09 | [-0.11- -0.07] | 0.000 |
| Gender | 0.05 | [0.01- 0.10] | 0.012 |
| Video game time | -0.05 | [-0.06- -0.03] | 0.000 |
| Sleep deprivation over device |  |  |  |
| Video game time | 0.22 | [0.16- 0.29] | 0.000 |

Note: Model explored video game time effect on GPA mediated by sleep deprivation and adjusted with age and gender variables; CI = Confidence interval.

Table c: Mediation analysis of sleep deprivation in the association between cellphone time during weekends and Academic performance (GPA), using structural equation model.

| GPA (outcome) | Beta | [95% CI] | P value |
| --- | --- | --- | --- |
| Sleep deprivation | -0.12 | [-0.18- -0.06] | 0.000 |
| Age | -0.07 | [-0.09- -0.05] | 0.000 |
| Gender | 0.12 | [0.08- 0.16] | 0.000 |
| Cellphone time | -0.04 | [-0.05- -0.03] | 0.000 |
| Sleep deprivation over device |  |  |  |
| Cellphone time | 0.27 | [0.22- 0.33] | 0.000 |

Note: Model explored cellphone time effect on GPA mediated by sleep deprivation and adjusted with age and gender variables; CI = Confidence interval.
